# Disturbance of Oligodendrocyte Function Plays a Key Role in the Pathogenesis of Schizophrenia and Major Depressive Disorder

**DOI:** 10.1155/2015/492367

**Published:** 2015-02-01

**Authors:** Shingo Miyata, Tsuyoshi Hattori, Shoko Shimizu, Akira Ito, Masaya Tohyama

**Affiliations:** ^1^Division of Molecular Brain Science, Research Institute of Traditional Asian Medicine, Kinki University, 337-2 Ohno-higashi, Osaka-sayama, Osaka 589-8511, Japan; ^2^Department of Neuroanatomy, Kanazawa University Graduate School of Medical Sciences, Kanazawa, Ishikawa 920-5111, Japan; ^3^Department of Molecular Neuropsychiatry, Graduate School of Medicine, Osaka University, Suita, Osaka 565-0871, Japan; ^4^Osaka Prefectural Hospital Organization, Osaka 558-8558, Japan

## Abstract

The major psychiatric disorders such as schizophrenia (SZ) and major depressive disorder (MDD) are thought to be multifactorial diseases related to both genetic and environmental factors. However, the genes responsible and the molecular mechanisms underlying the pathogenesis of SZ and MDD remain unclear. We previously reported that abnormalities of disrupted-in-Schizophrenia-1 (DISC1) and DISC1 binding zinc finger (DBZ) might cause major psychiatric disorders such as SZ. Interestingly, both DISC and DBZ have been further detected in oligodendrocytes and implicated in regulating oligodendrocyte differentiation. DISC1 negatively regulates the differentiation of oligodendrocytes, whereas DBZ plays a positive regulatory role in oligodendrocyte differentiation. We have reported that repeated stressful events, one of the major risk factors of MDD, can induce sustained upregulation of plasma corticosterone levels and serum/glucocorticoid regulated kinase 1 (Sgk1) mRNA expression in oligodendrocytes. Repeated stressful events can also activate the SGK1 cascade and cause excess arborization of oligodendrocyte processes, which is thought to be related to depressive-like symptoms. In this review, we discuss the expression of DISC1, DBZ, and SGK1 in oligodendrocytes, their roles in the regulation of oligodendrocyte function, possible interactions of DISC1 and DBZ in relation to SZ, and the activation of the SGK1 signaling cascade in relation to MDD.

## 1. Introduction

Schizophrenia (SZ) is a chronic, severe, and disabling brain disorder of mostly unknown etiology and pathophysiology. To date, a number of linkage and association studies have shown that multiple genes, such as disrupted-in-Schizophrenia-1 (DISC1), are involved in the development of SZ [1–5]. The* DISC1* gene was found to be specifically disrupted by a t(1;11) (q42.1;q14.3) balanced translocation in a large Scottish pedigree with several major mental illnesses, such as SZ and bipolar affective disorder [6–8]. Previous studies showed that DISC1 and the DISC1 binding zinc finger (DBZ) have multiple functions for neuronal proliferation, migration, and differentiation during brain development and modulation of* Disc1* in rodents can cause behavioral changes through its dysfunction in neurons [1–5, 9–18]. Recently, both DISC1 and DBZ have been further detected in oligodendrocytes and implicated in regulating oligodendrocyte differentiation [19–21].

Multiple lines of evidence obtained by brain imaging, postmortem brain studies, and genetic association studies have implicated oligodendrocyte abnormalities and compromised white matter/myelin integrity in neuropsychiatric diseases including SZ, bipolar disease (BP), major depressive disorder (MDD), autism, and attention deficit hyperactivity disorder (ADHD), as well as in central nervous system (CNS) disorders with pronounced cognitive impairments, such as Alzheimer's disease [22, 23]. Further, the high cooccurrence of white matter/myelin diseases with SZ-like psychoses, such as multiple sclerosis, leukodystrophies, and velocardiofacial syndrome, suggests that oligodendrocytes and myelin dysfunction may play a key role in the pathogenesis of neuropsychiatric diseases [24–26]. However, neurobiological studies of these diseases have predominantly focused on neurons, and only a few reports have studied oligodendrocytes [27–29].

Furthermore, MDD is thought to be a multifactorial disease related to both environmental and genetic factors. However, the genes responsible and the pathogenesis of MDD at the molecular level remain unclear. Among many environmental factors, repeated stressful events are associated with the onset of MDD, and stress activates the hypothalamic-pituitary adrenocortical (HPA) system [30–34]. In fact, antidepressant treatment partly normalizes hyperactivity of the HPA axis in patients with depression [35]. Many clinical cases have demonstrated that elevated corticosterone levels trigger depressive symptoms. For example, patients with Cushing disease, in whom corticosteroids are excessively secreted, frequently exhibit depressive symptoms. Additionally, patients chronically treated with exogenous corticosteroids exhibit depressive symptoms referred to as steroid psychosis [36]. These findings show that sustained elevated levels of plasma corticosteroids are one of the causes of MDD.

However, until now, the molecular pathway affected by excess levels of plasma corticosteroids was unknown. Recently, molecules downstream of elevated plasma corticosteroids have been explored using water immersion restraint stress (WIRS) as a stressor [37]. In addition, we have confirmed that the same molecular pathway is activated in models of social isolation and social defeat stress (unpublished data). It should be noted that activation of this SGK1 pathway by plasma corticosterone occurs via oligodendrocytes, and several studies have shown a close relationship between the pathogenesis of MDD and oligodendrocytes [38–42].

Oligodendrocyte abnormalities are implicated in the pathogenesis of several psychiatric disorders. Furthermore, in recent years, we have shown that DISC1 and DBZ function in oligodendrocytes have some involvement in SZ pathogenesis and that SGK1 function in oligodendrocytes is closely related to MDD pathogenesis. Thus, in this review, we will focus on the role of DISC1, DBZ (Sections 2 and 3), and SGK1 (Section 4) in oligodendrocytes and their relation to major psychiatric disorders, especially SZ and MDD.

## 2. Involvement of Oligodendrocytes in Disrupted-in-Schizophrenia-1- (DISC1-) Related Psychiatric Disorders

A previous report showed that the DBZ protein, also known as ZNF365 or Su48, is a novel DISC1 binding protein with a predicted C2H2-type zinc-finger motif and coiled-coil domains [2]. DBZ is unique among more than 90 DISC1-binding proteins, because DBZ is specifically expressed in the CNS* in vivo*. In neuronal cells, DBZ regulates neurite extension via a DISC1-DBZ interaction in primary cultured neurons and PC12 cells [2]. In addition, cortical GABA neurons remain immature in DBZ knockout (KO) mice [43]. Moreover, an essential role for DBZ in the mitosis of cancer cell lines of peripheral origin has been reported [44].

A close relationship between neuronal DISC1 and DBZ has been established in the regulation of neural circuit formation. Furthermore, disruption of neural circuit formation, due to abnormalities of DISCI and DBZ, may cause major psychiatric disorders such as SZ. Both DISC and DBZ have been recently identified as being expressed in oligodendrocytes and are implicated in regulating oligodendrocyte differentiation [19, 20]. Taken together, these findings suggest that abnormalities in oligodendrocyte differentiation result in the expression of major psychiatric diseases [19, 20]. In the next section, we summarize the expression of DISC1 and DBZ in oligodendrocytes, their functional roles, and possible interactions of these two molecules in their regulation of oligodendrocyte function.

## 3. DISC1 and DBZ Have Opposite Regulatory Roles in Oligodendrocyte Differentiation

### 3.1. Both DISC1 and DBZ Are Expressed in Oligodendrocyte Lineage Cells

**Figure 1:**
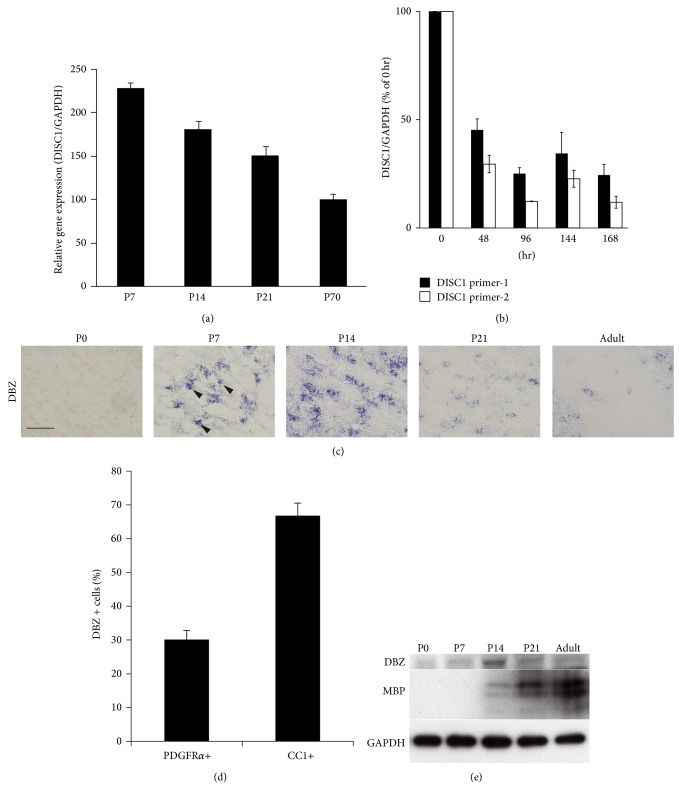
*DISC1* and* DBZ* mRNA is expressed in oligodendrocytes in the corpus callosum (CC) of the mouse brain. (a) Real-time PCR analysis of DISC1 in CC at the indicated stages. Data are expressed from at least three independent experiments. DISC1 mRNA levels at P7 were higher than at later time points. *P* < 0.01, as assessed via a one-way ANOVA followed by Tukey's test. (b) Primary cultured rat OPCs were harvested at indicated times after platelet-derived growth (PDGF) deprivation. mRNA was quantified by qRT-PCR. Data are expressed from at least three independent experiments. DISC1 mRNA levels at 0 h were higher than at later time points. *P* < 0.01, as assessed via a one-way ANOVA followed by Tukey's test. (c) Postnatal development of* DBZ* mRNA in the CC of mice from P0, P7, P14, P21, and adults.* DBZ* mRNA-expressing cells were first identified at P7. The number and intensity of the reaction of DBZ-positive cells reached to peak at P14, followed by its progressive reduction until adulthood. Scale bar: 50 *μ*m. (d) Quantification of the proportion of DBZ+/PDGFR*α*+ and DBZ+/CCI+ cells. Double labeling with* in situ* hybridization and immunohistochemical analysis of brain sections from P14 mice with the antisense RNA probe to* DBZ* and antibodies against Olig2, PDGFR*α*, and CC1, respectively. (e) Western blot analysis of DBZ and MBP in the CC at the indicated stages. DBZ protein expression peaked at P14 before MBP expression. (b) is adapted with permission from Hattori et al. [20] and (c)–(e) are adapted with permission from Shimizu et al. [19].

Previously, a double labeling technique for combined* in situ* hybridization and immunohistochemical analysis was adopted to elucidate whether DISC1 and DBZ were localized in oligodendrocytes in the mouse corpus callosum (CC), where oligodendrocyte lineage cells are abundant. These studies report DISC1 mRNA in the CC of postnatal day (P)14 and P70 mouse brains with a slight predominance in P14. Interestingly, these DISC1-positive cells simultaneously express adenomatous polyposis coli (APC), an oligodendrocyte marker [20]. Quantitative real-time polymerase chain reaction (qRT-PCR) analysis on the developmental expression of DISC1 mRNA in the CC of mice revealed the highest expression at P7, followed by a gradual decrease until adulthood (Figure 1(a)).

To confirm DISC1 mRNA expression levels during oligodendrocyte developmental stages, we have examined DISC1 expression during differentiation of oligodendrocyte precursor cells (OPC) to oligodendrocytes* in vitro* [20]. In order to accomplish this aim, primary cultured rat OPC cells were induced to differentiate into oligodendrocytes by depriving the culture medium of platelet-derived growth factor (PDGF). Interestingly, qRT-PCR analysis show reduced expression of DISC1 mRNA after PDGF deprivation (Figure 1(b)). Taken together, results from both* in vivo* and* in vitro* studies suggest that DISC1 is involved in the differentiation of oligodendrocytes.

Although DBZ mRNA has been shown to be expressed in the mouse CC, the pattern of postnatal DBZ mRNA expression is different from that of DISC1 [19, 20]. For one, DBZ mRNA-expressing cells have not been detected in the CC of P0, but many positive cells appear at P7 and even peak at P14 (Figure 1(c)). The number of mRNA-expressing cells can be observed to decrease thereafter until adulthood (Figure 1(c)). Furthermore, most Olig2- (a marker of both OPCs and oligodendrocytes-) immunoreactive cells in the CC of P14 mice express DBZ mRNA [19]. These DBZ mRNA-expressing cells can be visualized as being colabeled with the OPC marker, PDGF receptor alpha (PDGFR*α*), or the oligodendrocyte marker, CC1. Approximately 30% of DBZ mRNA expressing cells exhibit PDGFR*α*, whereas approximately 70% express CC1 (Figure 1(d)) [19]. These findings indicate that DBZ mRNA is expressed in oligodendrocyte lineage cells and its expression is higher at later developmental stages.

qRT-PCR analysis revealed an increase in DBZ mRNA during the first week after birth, although the increase was not statistically significant. However, during the second week, DBZ mRNA expression significantly increased, followed by a gradual decrease until adulthood [19]. The time course of the DBZ protein has also been examined by western blot analysis, which revealed that DBZ expression peaks at P14, similar to DBZ mRNA expression (Figure 1(e)).

The development of DBZ in the mouse CC has been compared to that of myelin basic protein (MBP) (a major constitution of the myelin sheath). As shown in Figure 1(e), DBZ expression peaks before that of MBP; this suggests a regulatory role for DBZ in oligodendrocyte differentiation and/or myelin formation.

### 3.2. DISC1 in an Oligodendrocyte Lineage Cell Negatively Regulates Oligodendrocyte Differentiation

**Figure 2:**
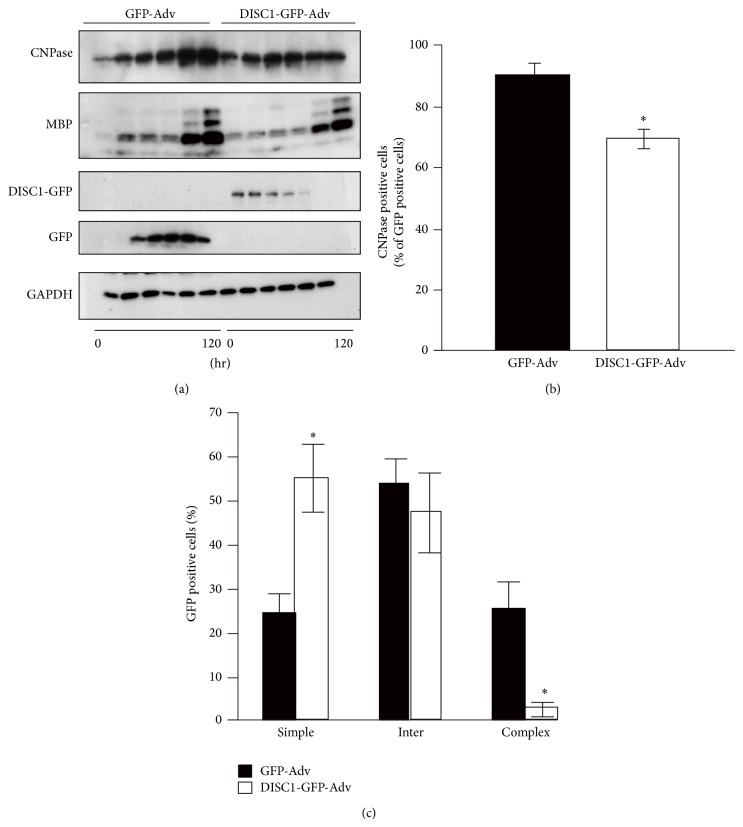
DISC1 overexpression inhibits oligodendrocyte differentiation. (a) Primary cultured rat OPCs infected with GFP-Adv or DISC1-GFP-Adv were lysed at 0, 24, 48, 72, 96, and 120 hours after PDGF deprivation and subjected to western blot analysis. (b) Primary cultured rat OPCs were immunostained with anti-GFP and anti-CNPase antibodies. The percentage of CNPase positive cells relative to the total number of GFP positive cells is shown. Infected cells from three experiments were analyzed. ^*^ *P* < 0.05 versus GFP-Adv. Scale bar = 100 *μ*m. (c) Primary cultured rat OPCs were immunostained with anti-GFP and anti-*β*-tubulin antibodies for morphological observation. Infected cells from three independent cultures were classified according to their morphology (simple, intermediate, or complex) and quantified. The percentage of cells within each category, relative to the total number of GFP positive cells, is shown. ^*^ *P* < 0.05 versus GFP-Adv. Scale bar = 50 *μ*m. (a)–(c) are adapted with permission from Hattori et al. [20].

In primary cultured rat OPCs induced to differentiate 12 h after DISC1 overexpression, expression of the myelin genes, 2′,3′-cyclic nucleoside-3′phosphodiesterase (CNPase) and MBP, were observed to decrease at both the mRNA and protein level, compared with control-infected cells (GFP-Adv) (Figure 2(a)) [20]. To confirm the reduced CNPase in DISC1 overexpressing cells, the ratio of GFP-expressing cells with CNPase expression in GFP-Adv or DISC1-Adv infected cells was evaluated by immunohistochemistry. Using this approach, the percentage of CNPase-immunoreactive cells was observed to decrease 96 h after PDGF deprivation, following DISC1 overexpression (about 70%) compared with control cells (about 91%) (Figure 2(b)).

The morphological transformation of cells by DISC1 overexpression has also been examined 96 h after PDGF deprivation. The morphology of infected cells can be classified as simple, intermediate, or complex. In these studies, about 25% of control cells were observed to have a complex morphology, characterized by the presence of several interlaced fine branches indicative of advanced differentiation. About 25% of cells, including undifferentiated cells, showed a simple morphology defined by primary branches and the absence of secondary and tertiary processes. In contrast, only 2-3% of DISC1 overexpressing cells displayed a complex morphology and about 56% showed a simple morphology (Figure 2(c)). Thus, overexpression of DISC1 results in a reduction of matured oligodendrocytes, suggesting that decrease in endogenous DISC1 expression in OPC cells upon PDGF deprivation promote oligodendrocyte differentiation.

### 3.3. DISC1 Knockdown Promotes Oligodendrocyte Differentiation

**Figure 3:**
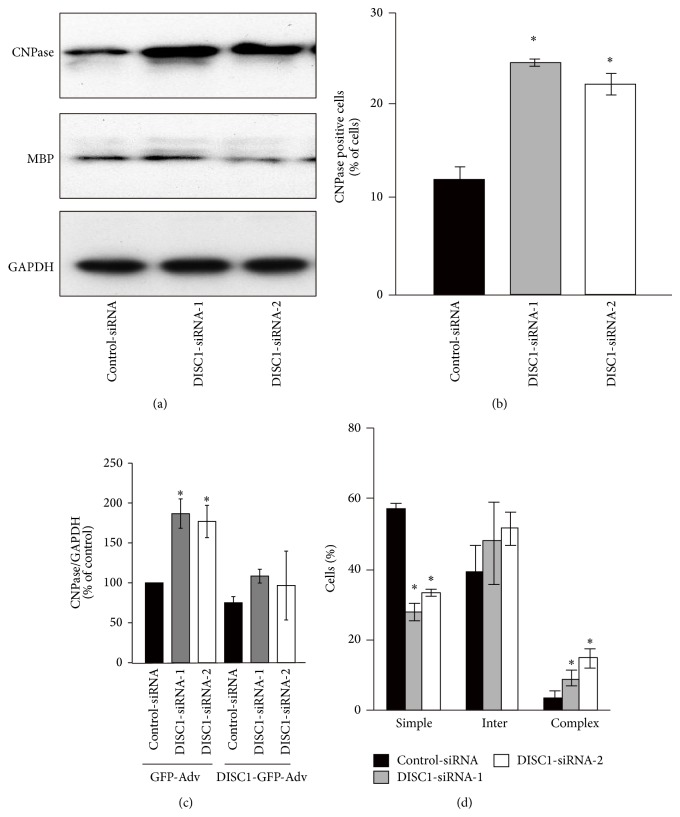
DISC1 knockdown promotes oligodendrocyte differentiation. (a) Primary cultured rat OPCs transfected with control-siRNA, DISC1-siRNA-1, or DISC1-siRNA-2 were lysed 72 hours after siRNA transfection and analyzed by western blotting. (b, d) Primary cultured rat OPCs were transfected with control-siRNA, DISC1-siRNA-1, or DISC1-siRNA-2 and cultured in medium containing PDGF for 72 hours then fixed for immunostaining. Primary cultured rat OPCs were immunostained with anti-CNPase-antibody (b) or anti-*β*-tubulin antibody (d) and analyzed as described here. ^*^ *P* < 0.05 versus control-siRNA. Scale bars = 50 *μ*m. (c) DISC1 knockdown mediated increase of CNPase mRNA was rescued by overexpression of DISC1. Primary cultured rat OPCs were infected with GFP- or DISC1-GFP-Adv 24 hours after control- or DISC1-siRNA transfection. Forty-eight hours after the infection, mRNA level of CNPase was quantified by qRT-PCR. Data are expressed as mean ± SEM of at least three independent experiments. ^*^ *P* < 0.05 versus control-siRNA and GFP-Adv. (a)–(d) are adapted with permission from Hattori et al. [20].

Suppression of DISC1 expression by small interference RNA (siRNA) targeting* DISC1* mRNA (DISC1-siRNA-1 or DISC1-siRNA-2) has been shown to increase both mRNA and protein levels of CNPase (Figure 3(a)) [20]. In these studies, a tendency towards increased* MBP* mRNA expression was also observed (Figure 3(a)) [20].* In vitro* immunostaining of oligodendrocyte lineage cells have revealed that* DISC1* knockdown causes a two-fold increase in the percentage of CNPase-positive cells (*DISC1* knockdown: about 25% of the total; control about 12% of the total), compared with control-siRNA treatment (Figure 3(b)). Interestingly, the co-expression of* DISC1* siRNA and human DISC1-GFP can rescue the increased CNPase expression (Figure 3(c)).

Finally, the morphological transformation of cells transfected with siRNA has been examined by immunohistochemistry for *β*-tubulin 72 h after transfection. About 58% of control cells show simple morphology and only 4% display complex morphology (Figure 3(d)). However,* DISC1* knockdown has been found to decrease the cells that exhibit a simple morphology (about 28% of total cells), and increase cells with a complex morphology (about 10% of total cells) (Figure 3(d)). These findings suggest that endogenous DISC1 in an oligodendrocyte lineage cell negatively regulates oligodendrocyte differentiation. This negative regulatory role of DISC1 was confirmed by the overexpression of truncated DISC1, which was predicted to function in a dominant negative fashion, using primary cultures of rat oligodendrocytes [20].

### 3.4. Conclusion Regarding the Function of DISC1 on Oligodendrocyte Differentiation

Taken together, the findings mentioned above show that endogenous DISC1 negatively regulates the differentiation of oligodendrocyte precursor cells into oligodendrocytes during the early postnatal stage (see Figure 9).

### 3.5. DBZ Deficiency Delays Myelination during the Postnatal Period; However, Myelination Recovers by Adulthood

**Figure 4:**
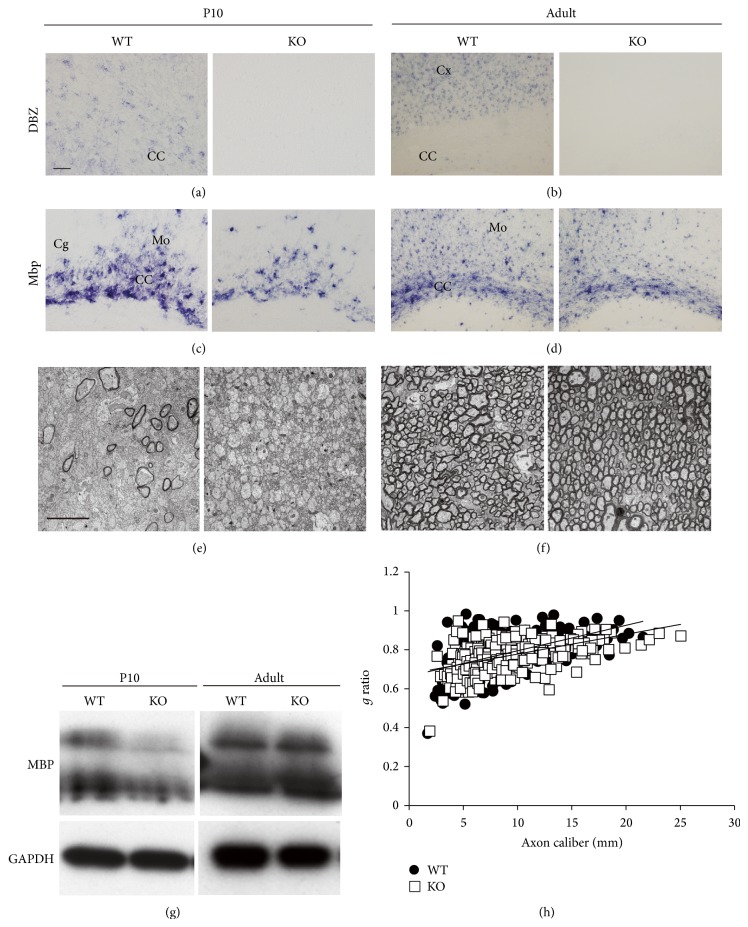
Myelination in the CC of DBZ-KO mice is delayed during the postnatal period but recovers by adulthood. (a–d)* In situ* hybridization analyses of WT and* DBZ*-KO littermates at P10 (a, c) or adulthood (b, d) with the antisense RNA probes to* DBZ* (a, b) and* Mbp* (c, d) mRNA. The number of cells in the CC of* DBZ*-KO mice expressing* Mbp* mRNA was significantly reduced at P10 compared with age-matched WT mice, but it was recovered to WT levels by adulthood. Cg: cingulate gyrus, Mo: motor area. (e, f) Electron microscopic analyses of the CC in P10 (e) or adult (f). (g) Western blot analyses of MBP protein in the CC of WT or* DBZ*-KO mice at P10 or adulthood. (h) Scatter plot of *g*-ratio values in the CC of WT or* DBZ*-KO mice at adulthood. Four hundred and four axons from four animals were analyzed for each group. Scale bar: (a–d) 50 *μ*m, (e, f) 5 *μ*m. (a)–(h) are adapted with permission from Shimizu et al. [19].

It has been shown that the number of* Mbp* mRNA-expressing cells in the CC of* DBZ*-KO mice is significantly reduced at P10, but that number recovers to wild type (WT) levels by adulthood (Figures 4(a)–4(d)). Myelin-associated glycoprotein (*Mag*), a transmembrane glycoprotein expressed in myelinating oligodendrocytes, shows a similar expression pattern as* Mbp* [19].

Subsequent immunohistochemical analyses demonstrated a striking reduction in the immunoreactivity of both myelin markers at P10 in* DBZ*-KO mice compared with age-matched WT controls [19]. There were also fewer immunoreactive nerve fibers passing the CC of* DBZ*-KO mice and their projections to the cingulate (Cg) or motor area (Mo) were shorter compared with WT mice. Moreover, these studies reported fewer MBP- or MAG-positive oligodendrocytes in the motor cortex of* DBZ*-KO mice. However, no such differences were observed between adult* DBZ*-KO and WT mice [19]. Western blot analysis also supported the finding of reduced MBP protein expression in the CC of* DBZ*-KO mice compared with WT mice at P10 but not at adulthood (Figure 4(g)).

Alterations of myelin fibers in* DBZ*-KO mice have also been revealed at the ultrastructural level. At P10, most nerves in the CC of* DBZ*-KO mice are observed to be unmyelinated and small in diameter (Figure 4(e)). This result is in sharp contrast to the number of large diameter myelinated nerves in WT (Figure 4(e)). However, similar to the difference in MBP and MAG expression, the reduced myelination in the CC of P10* DBZ*-KO mice is recovered by adulthood (Figure 4(f)). In addition, the generation of myelinated axons in adult* DBZ*-KO and WT mice is comparable. In other words, there are no detectable differences of myelinated axon generation between* DBZ*-KO and WT mice in the CC (Figure 4(h)).

These data reveal that delayed myelination in* DBZ*-KO mice at P10 can recover by adulthood, suggesting that DBZ plays a regulatory role in oligodendrocyte differentiation and/or myelin formation.

### 3.6. Increased Immature Oligodendrocytes in DBZ-KO Mice

**Figure 5:**
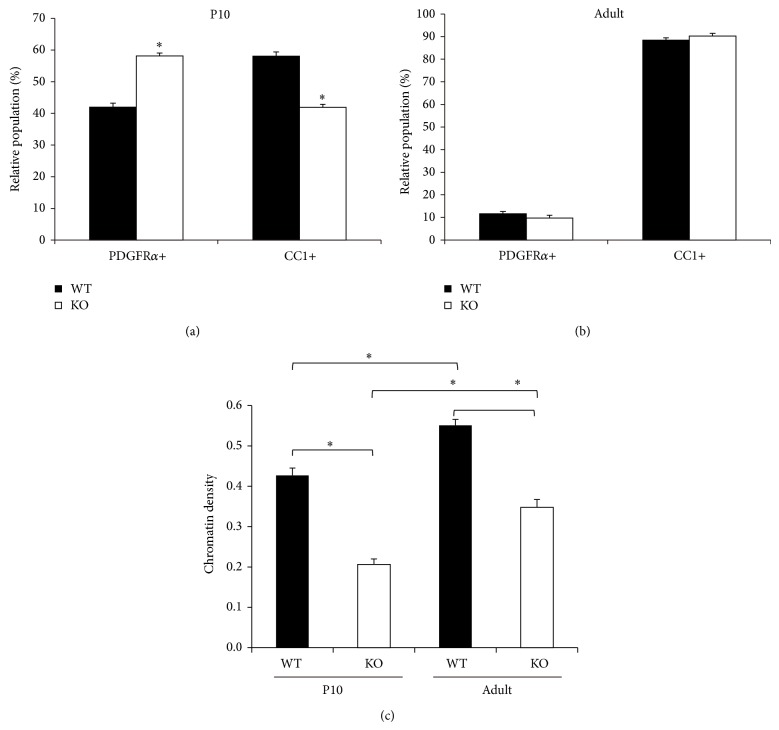
Immature oligodendrocytes are increased in* DBZ*-KO mouse. (a, b) Immunohistochemistry with antibodies against PDGFR*α* and CC1 on the CC sections from WT and* DBZ*-KO littermates at P10 or adult. Quantification of the proportion of PDGFR*α*+ and CC1+ cells at P10 (a) and adulthood (b). (c) Chromatin density was calculated as the ratio of electron dense chromatin area to the cell nucleus area using ImageJ software. One hundred and forty oligodendrocytes from four animals were analyzed for each group. ^*^ *P* < 0.0001 by Student's *t*-test following two-way-ANOVA. (a)–(c) are adapted with permission from Shimizu et al. [19].

An increase of immature oligodendrocytes in the* DBZ*-KO mice CC has been confirmed by immunohistochemistry, using PDGF*α* or CC1, which are markers for OPC or mature oligodendrocytes, respectively. More specifically, PDGF*α*-positive cells (OPCs) to total lineage cells appear higher in* DBZ*-KO mice (*DBZ*-KO mice: about 60%; WT mice: about 42%), while that of CC1-positive cells (matured oligodendrocytes) is lower in* DBZ*-KO mice (*DBZ*-KO mice: about 42%; WT: about 60%) at P10 (Figure 5(a)). However, no such differences are observed between adult* DBZ*-KO and WT mice (Figure 5(b)). These results suggest that DBZ plays a positive role in the development of OPCs into mature oligodendrocytes at P10 when oligodendrocyte differentiation and myelination are active.

The above light microscopic observations have also been confirmed by electron microscopy. Oligodendrocytes have been categorized into the following three types based on their ultrastructural features [45]: (1) light-oligodendrocytes (LO) or immature cells with evenly distributed cytoplasmic organelles; (2) dark-oligodendrocytes (DO) or mature cells with large amounts of chromatin and scanty cytoplasm, containing lamellar rough endoplasmic reticulum (ER); and (3) medium-shade-oligodendrocytes (MO) or premature cells with a well-developed Golgi apparatus, showing the intermediate characteristics of LO and DO. Although there is no obvious difference in the ultrastructural features of cells in each category, the proportions of LOs and DOs in adult* DBZ*-KO mice are different from those in WT mice [19]. Similar to adult mice, the proportion of LOs in P10* DBZ*-KO mice are larger compared with WT mice, while the proportion of DOs is smaller in P10* DBZ*-KO mice [19]. In addition, some of the cells categorized as DOs in P10* DBZ*-KO mice have been reported to exhibit less-matured characteristics (i.e., MO-resembling) [19]. Heterochromatin density in adult mice has been reported to be higher than in P10 mice, irrespective of genotype (Figure 5(c)). Moreover, heterochromatin density is significantly lower in* DBZ*-KO mice than in WT mice at both P10 and adulthood (Figure 5(c)). Thus, electron microscopic analysis of oligodendrocytes reveals that oligodendrocytes with immature features are more abundant in* DBZ*-KO mice at both P10 and adulthood.

Both immunohistochemical and electron microscopic observations showed consistent defects in oligodendrocyte maturation/myelination in* DBZ*-KO mice at P10 [19]. On the other hand, results for adult mice differ depending on the methodology employed, that is, light versus electron microscope. However, it is probable that there is little, if any, oligodendrocyte maturation/myelination defect in adult* DBZ*-KO mice.

### 3.7. DBZ Knockdown Does Not Affect Oligodendrocyte Proliferation* In Vitro*

To explore the potential role of endogenous DBZ in OPC proliferation, effects of* DBZ* knockdown have been examined by the siRNA method. Using this method, it has been determined that there is no significant difference in BrdU incorporation rates in OPCs transfected with* DBZ*-siRNA or control-siRNA, suggesting that endogenous DBZ plays no major role in OPC proliferation* in vitro* [19].

### 3.8. DBZ Is Transiently Upregulated* In Vitro* during Oligodendrocyte Differentiation before Myelin Marker Expression

**Figure 6:**
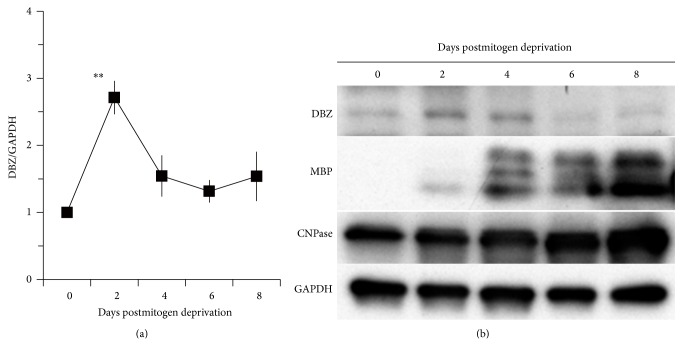
DBZ is transiently upregulated* in vitro* during oligodendrocyte differentiation before myelin marker expression. (a) Primary OPC were isolated from rat postnatal cortex and differentiated by mitogen withdrawal. Total RNA was prepared from the cells 0, 2, 4, 6, or 8 days after mitogen withdrawal.* DBZ* mRNA expression was evaluated using qRT-PCR, with normalization to GAPDH. Data normalized to either day 0. ^*^ *P* < 0.05, ^**^ *P* < 0.01 versus day 0 or +*P* < 0.05, and ++*P* < 0.01 versus day 2 by Dunnett's test following one-way ANOVA, *n* = 2–4. (b) Western blot analyses of DBZ, MBP and CNPase from cells 0, 2, 4, 6, or 8 days after mitogen withdrawal. (a) and (b) are adapted with permission from Shimizu et al. [19].

DBZ mRNA expression, and myelin-markers such as* Mbp*,* Mag*, and* CNPase*, during* in vitro* oligodendrocyte differentiation has been examined. Specifically, these studies found that* Mbp* and* Mag* mRNA expression sharply increases in a time-dependent fashion after mitogen deprivation, indicating the progression of differentiation [19].* CNPase* mRNA also appears to increase in a time-dependent manner after mitogen deprivation, although this increase is more gradual [19]. In contrast to the increase in expression of myelin-related protein (which lasts at least 8 days after mitogen deprivation),* DBZ* expression has been observed to peak 2 days after mitogen deprivation and then return to basal levels (Figure 6(a)). DBZ, MBP, and CNPase protein expression follow a similar time course, as determined by western blotting (Figure 6(b)).

Thus, these* in vitro* findings are consistent with* in vivo* results showing that DBZ expression is transiently upregulated during oligodendrocyte differentiation before the expression of oligodendrocyte differentiation markers.

### 3.9. DBZ Knockdown Results in Delayed Oligodendrocyte Differentiation* In Vitro*

**Figure 7:**
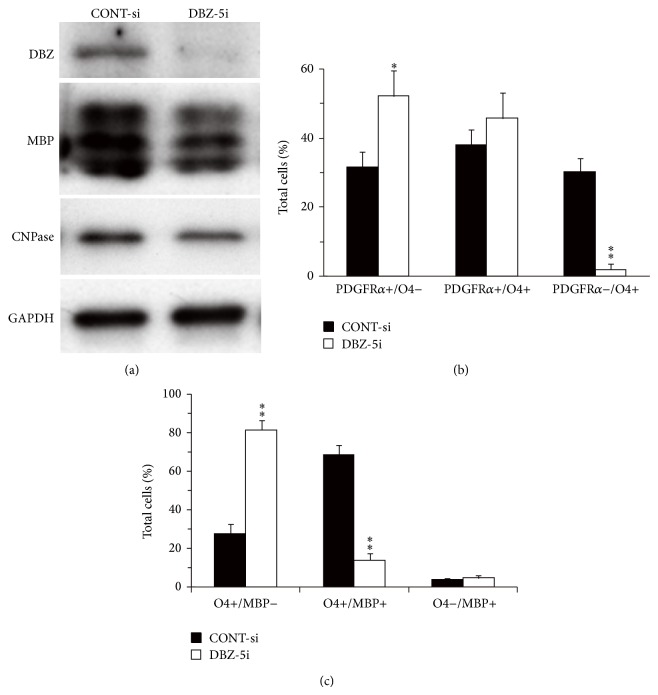
DBZ knockdown results in an* in vitro* delay in oligodendrocyte differentiation. Primary cultures of OPC were transfected with DBZ-5i or CONT-si 24 h before deprivation of PDGF and bFGF to induce differentiation. (a) Lysates from primary cultured OPCs were prepared from cells harvested 48 h after mitogen deprivation and expression of DBZ, MBP, and CNPase was assessed by western blotting. (b) Primary cultured OPCs were immunostained with PDGFR*α* and O4 antibodies 48 h after mitogen deprivation. Quantification of the proportion of PDGFR*α*+/O4−, PDGFR*α*+/O4+, and PDGFR*α*−/O4+ cells. (^*^ *P* < 0.05, ^**^ *P* < 0.01 versus WT, *n* = 3) (c) Primary cultured OPCs were immunostained with O4 and MBP antibodies 96 h after mitogen deprivation. Quantification of the proportion of O4+/MBP−, O4+/MBP+, and O4−/MBP+ cells f (^**^ *P* < 0.01 versus WT, *n* = 3). For (b) and (c), more than 400 cells in total from three independent cultures were counted. (a)–(c) are adapted with permission from Shimizu et al. [19].

Knockdown of* DBZ* by siRNA (DBZ-5i) has been found to reduce the mRNA expression of* Mbp*,* Mag*, and* CNPase* [19]. Moreover, a decrease in MBP and CNPase expression upon* DBZ*-siRNA treatment has also been confirmed at the protein level using western blot analysis (Figure 7(a)).

Morphological observations have revealed that 48 h after mitogen deprivation about 32% of cells in control-siRNA treated cultures are PDGFR*α* (marker for the most immature cells)−/O4 (marker for immature cells which are more differentiated than PDGFR*α* positive cells)+ immature oligodendrocytes, whereas PDGF*α*+/O4− cells, which are considered less differentiated, are about 33% of total cells (Figure 7(b)) [19]. In contrast, in* DBZ*-siRNA-treated cultures, very few PDGFR*α*−/O4+ cells can be observed (about 3%) and a significantly higher proportion of PDGFR*α*+/O4− cells (about 55%) has been reported. When analyzed 96 h after mitogen deprivation, about 70% of cells treated with control-siRNA differentiate to the O4+/MBP (marker for matured oligodendrocytes)+ stage; however, only 15% of cells can be identified at this stage when* DBZ* is knocked down (Figure 7(c)) [19]. Indeed, less differentiated O4+/MBP− cells are more abundant in* DBZ*-siRNA-treated cultures compared with control-siRNA-treated cultures (about 83% versus 29%) (Figure 7(c)) [19]. Thus, more immature oligodendrocytes are observed in* DBZ*-siRNA-treated cultures at both 48 and 96 h after mitogen deprivation. These findings indicate that knockdown of endogenous* DBZ* delays the progression of sequential events leading to oligodendrocyte differentiation.

**Figure 8:**
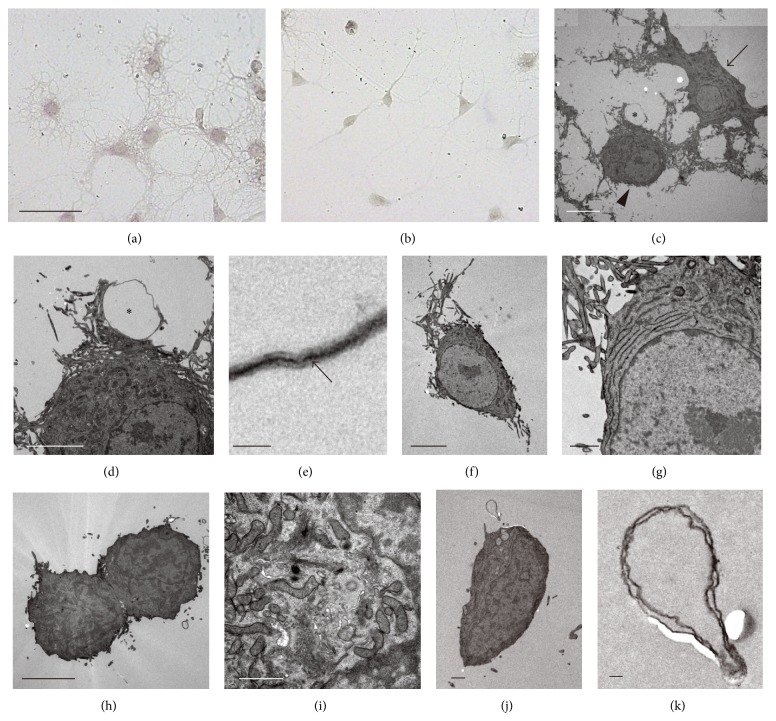
Effect of* DBZ* knockdown on oligodendrocytes cell morphology. Morphological features of primary cultured oligodendrocytes treated with CONT-si or DBZ-5i as determined by light microscopy (a, b) or electron microscopy (c–k). Micrographs of CONT-si-treated oligodendrocytes (a, c–g) or DBZ-5i-treated oligodendrocytes (b, h–k). CONT-si-treated oligodendrocytes had morphological characteristics of Type 2 or Type 3 cells corresponding to immature oligodendrocytes or mature oligodendrocytes, respectively, whereas DBZ-5i-treated oligodendrocytes were largely characterized by Type 1 corresponding to OPCs. Scale bar: (a, b) 50 *μ*m, (c) 10 *μ*m, (d, f, g, h) 5 *μ*m, (e) 0.1 *μ*m, (i, j) 1 *μ*m, (k) 0.1 *μ*m. (a)–(k) are adapted with permission from Shimizu et al. [19].

Developmental stages of cultured oligodendrocytes can be morphologically categorized into Type 1, Type 2, or Type 3 stages, corresponding to OPCs (Type 1), immature oligodendrocytes (Type 2), or matured oligodendrocytes (Type 3), respectively [46]. DBZ-siRNA-treated oligodendrocytes largely exhibit Type 1 morphology (Figure 8(b)), while most control oligodendrocytes differentiate to cells with Type 2 or Type 3 morphological characteristics (Figure 8(a)). An ultrastructural analysis of control oligodendrocytes displayed (Figure 8(c), arrowhead) cells rich in organelles, including regularly layer-arranged ERs, mitochondria, and polysomes (Figures 8(f) and 8(g)). The ring-like structure that is characteristic of Type 3 processes (Figures 8(c) and 8(d), asterisk) is typical of myelin formation, because of the dense line identified at the center of these processes (Figure 8(e), arrow). Type 2 cells (Figure 8(c), arrow) have abundant cytoplasm-containing ERs, mitochondria, and Golgi apparatuses. Furthermore, much of the nuclear chromatin in Types 2 and 3 cells tend to clump and lie beneath the nuclear envelop. A portion of control cells belong to the Type 2 stage (Figures 8(c), 8(d), 8(e), and 8(g)). In contrast, oligodendrocytes in* DBZ*-knocked down cells are quite different from those of matured cell types. Compared to control oligodendrocytes,* DBZ*-knocked down cells are smaller, exhibit fewer short processes (Figures 8(h) and 8(j)), and display a cytoplasmic Golgi apparatus that is well-developed (Figure 8(i)). Unlike control cells, myelination of the processes and nuclear chromatin condensation cannot be observed in oligodendrocytes where* DBZ* has been knocked down (Figures 8(h), 8(j), and 8(k)).

### 3.10. Conclusion of DBZ Function on Oligodendrocyte Differentiation

**Figure 9:**
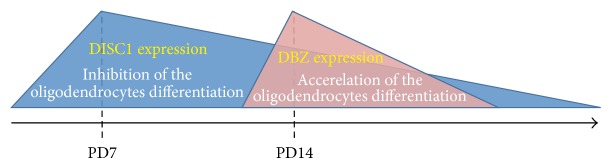
Interaction between DISC1 and DBZ on the oligodendrocytes development.

Considering the above-discussed* in vitro* findings, together with* in vivo* studies, it can be concluded that endogenous DBZ has a positive regulatory role in oligodendrocyte differentiation (Figure 9) [19, 20].

### 3.11. Possible Functional Relationship between DBZ and DISC1 in Oligodendrocytes

As discussed above, the highest expression of DISC1 in oligodendrocytes is already identifiable at P7, when DBZ expression is faint.* In vitro* as well as* in vivo*, the highest expression of* DISC1* mRNA has been found just after mitogen deprivation. DISC1 inhibits the differentiation of oligodendrocytes because (1) DISC1 overexpression inhibits the expression of myelin-related markers such as* MBP* and* CNPase* and decreases the number of cells with matured oligodendrocyte morphology, and (2) knockdown of endogenous DISC1 increases the expression of myelin-related markers and the number of cells with matured oligodendrocyte morphology. Thus, DISC1 may inhibit oligodendrocyte differentiation during early postnatal stages (Figure 9).

After P7, DBZ expression appears to reach its peak at P14, before MBP expression. In the CC of* DBZ*-KO mice, it has been reported that myelination is delayed during the early postnatal period. DBZ is also transiently upregulated during rat oligodendrocyte differentiation* in vitro* before myelin marker expression. Furthermore,* DBZ* knockdown results in a decreased expression of myelin-related markers and a low number of cells with mature characteristics but with no effects on the proliferation of oligodendrocyte precursor cells. These findings strongly indicate that DBZ positively regulates oligodendrocyte differentiation and myelination during the early postnatal stage (Figure 9). Since the peak of DISC1 expression in oligodendrocytes precede that of DBZ, it is likely that inhibition of the oligodendrocytes differentiation by DISC1 is released by DBZ.

### 3.12. Regulatory Pathway of Oligodendrocyte Lineage Cells by DISC1 and DBZ

**Figure 10:**
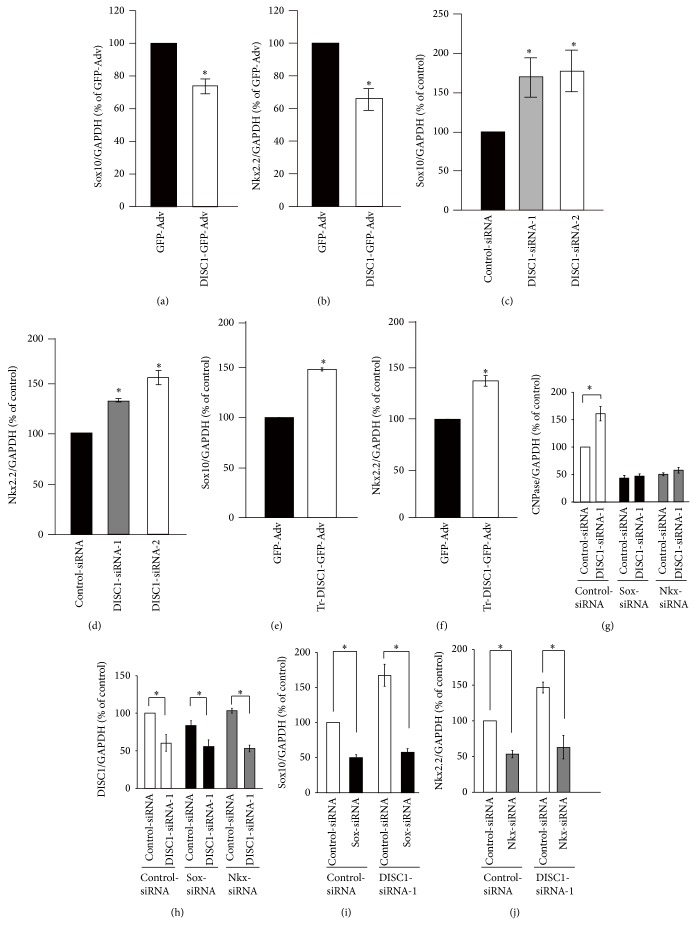
Involvement of Sox10 and/or Nkx2.2 in the regulatory pathway of oligodendrocyte differentiation by DISC1. (a, b) Expression of Sox10 and Nkx2.2 mRNA were decreased by DISC1 overexpression. Oligodendrocyte precursor cells were infected with GFP-Adv or DISC1-GFP-Adv for 12 hours and induced to differentiate by depriving PDGF for 36 hours. (c, d) Expression of Sox10 and Nkx2.2 mRNA were increased by DISC1 knockdown. Oligodendrocyte precursor cells were transfected with control-siRNA, DISC1-siRNA-1 or DISC1-siRNA-2 and cultured in medium containing PDGF for 48 hours. (e, f) Expression of Sox10 and Nkx2.2 mRNA were increased by truncated DISC1 overexpression. Oligodendrocyte precursor cells were infected with GFP-Adv or trDISC1-GFP-Adv for 12 hours and induced to differentiate by PDGF deprivation for 36 hours. (g–j) DISC1 knockdown mediated increase of CNPase mRNA was inhibited by a simultaneous knockdown of either Sox10 or Nkx2.2. Oligodendrocyte precursor cells were co-transfected with control-siRNA or DISC1-siRNA-1 and sox10-siRNA or nkx2.2-siRNA and cultured in medium containing PDGF for 24 (h) or 48 hours (g, i, j). mRNA quantification was performed 48 hours after adenovirus infection (a, b, e, f) or siRNA transfection (c, d, g, i, j) or 24 h hours after siRNA transfection (h) by qRT-PCR. Data are expressed as mean ± SEM of at least three independent experiments. ^*^ *P* < 0.05 versus GFP-Adv (a, b), ^*^ *P* < 0.05 versus control-siRNA (c, d), and ^*^ *P* < 0.05 (e–h). (a)–(j) are adapted with permission from Hattori et al. [20].

It has been shown that DISC1 overexpression can cause significant decreases of* Sox10* and* Nkx2.2* mRNA expression (Figures 10(a) and 10(b)). Furthermore, knockdown of endogenous* DISC1* has been shown to result in enhanced expression of* Sox10* and* Nkx2.2*, which have a positive regulatory role on oligodendrocyte differentiation (Figures 10(c) and 10(d)). Truncated forms of* DISC1* also increase* Sox10* and* Nkx2.2* expression (Figures 10(e) and 10(f)). However,* DISC1* knockdown has no effect on* Olig1* and* Olig2*, both of which play similar roles in oligodendrocyte differentiation. Similarly,* DISC1* knockdown has no effect on* Id2* and* Id4*, which negatively regulate oligodendrocyte differentiation.

**Figure 11:**
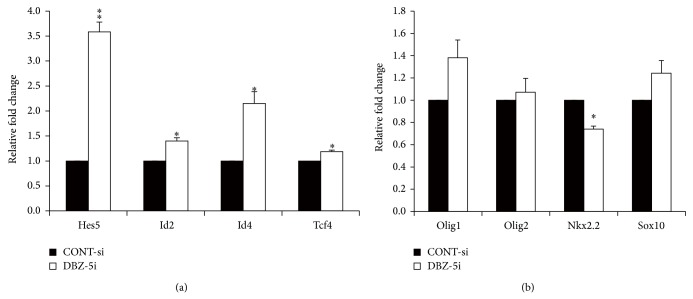
DBZ knockdown alters expression of transcription factors involved in OL differentiation. Primary cultures of OPCs were transfected with DBZ-5i or CONT-si 24 h before PDGF and bFGF deprivation to induce differentiation. Total RNA was prepared 24 h after mitogen deprivation. (a, b) The effect of* DBZ* knockdown on the expression of well-characterized transcription factors was examined using qRT-PCR. (a) Expression of the transcription factor negatively regulating oligodendrocyte differentiation. (b) Expression of the transcription factor positively regulating oligodendrocyte differentiation. ^**^ *P* < 0.01; ^*^ *P* < 0.05 versus CONT-si (*n* = 3). (a) and (b) are adapted with permission from Shimizu et al. [19].

The effect of simultaneously knocking down* Sox10* or* Nkx2.2* has been investigated in the primary culture of rat oligodendrocytes treated with* DISC1*-siRNA. More specifically, the promotion of oligodendrocyte differentiation by* DISC1* knockdown is inhibited (Figure 10(g)). The effect of* DISC1*-siRNA on its target mRNA is not altered when either* Sox10*-siRNA or* Nkx*-siRNA was cotransfected (Figure 10(h)). Similarly,* DISC1*-siRNA cotransfection does not alter the effect of* Sox*-siRNA or* Nkx*-siRNA (Figures 10(i) and 10(j)). Therefore, these results suggest that DISC1 negatively regulates oligodendrocyte differentiation by acting upstream of* Sox10* and/or* Nkx2.2* to suppress their expression. After mitogen deprivation,* DBZ*-siRNA treated primary cultures demonstrate a strong increase in the expression of transcription factors that negatively regulate oligodendrocytes such as* Hes5*,* Id2*,* Id4*, and* Tcf4* (Figure 11(a)). In contrast, the expression of transcription factors, such as Olig1, Olig2, Nkx2.2, and Sox10, which have a positive regulatory role on oligodendrocyte differentiation, are either unchanged or decreased when endogenous DBZ is knocked down (Figure 11(b)).

### 3.13. Conclusion about DISC1 and DBZ Functions in Oligodendrocytes in the Pathogenesis of SZ

Considering these findings, it is thus probable that during the early postnatal stage, oligodendrocyte expression of DISC1 is activated in order to inhibit oligodendrocyte differentiation. However, starting at P7 the expression of DISC1 in oligodendrocytes gradually decreases. This decrease is accompanied by an increase in the expression of DBZ, which acts to promote oligodendrocyte differentiation [19, 20].

Overall, DISC1 dysfunction may cause impaired differentiation of oligodendrocytes by affecting Sox10 and/or Nkx2.2 expression and consequently contribute to the pathophysiology of psychiatric disorders. Improper myelination of neuronal axons, resulting from impaired oligodendrocyte differentiation, may lead to defective neuronal communication, a likely component in the mechanistic background of “structural disconnectivity,” suggested in the pathophysiology of psychiatric disorders [47, 48].

DBZ, which we have identified as a brain-specific DISC1 binding protein, is identical to Su48 [44]. Su48 is a centrosome-associated protein that plays an essential role in the mitosis of cancer cell lines of peripheral origin by forming a complex with Nde1 [49]. Consistent with intracellular localization of overexpressed Su48 in cancer cell lines, we have observed forced centrosomal localization of DBZ in primary cultured neurons [2]. These results raise the possibility of a role for DBZ in OPC proliferation. However, the results of our BrdU incorporation assay indicate that the DBZ does not play a role in OPC proliferation [19].

Compromised white matter/myelin integrity has been reported in neuropsychiatric diseases including SZ, BP, and MDD [28, 37, 39–41, 50–52]. An increased abundance of rare nonsynonymous mutations in DBZ has been reported in patients with SZ, although association studies and gene expression analyses of DBZ with SZ and BP have yielded negative findings [53, 54]. Moreover, our preliminary behavioral study revealed an increase in anxiety in DBZ-KO mice [55].

Therefore, DISC1 and/or DBZ abnormalities are unlikely to be coincidental for some roles in the compromised white matter/myelin integrity of these psychiatric disorders.

## 4. Oligodendrocytes Play a Principal Role on the Occurrence of Stress-Induced MDD

Repeated stressful events are known to be closely associated with MDD onset [56–58]. Furthermore, stress activates the hypothalamic-pituitary-adrenocortical (HPA) system by elevating plasma cortisol levels. However, little is known about the related downstream molecular pathway [59–61]. In this section, we review our attempts to elucidate the molecular pathway induced by elevated plasma corticosterone levels by using repeated WIRS [37].

### 4.1. Expression of Serum/Glucocorticoid Regulated Kinase 1 (Sgk1) mRNA and the Active Form of SGK1 Protein Are Upregulated in Oligodendrocytes after Repeated Exposure to WIRS

Sgk1 is a serine/threonine kinase activated by the PI3K signal, and Sgk1 gene has a glucocorticoid responsive element on the promoter region [62–64]. In addition to oligodendrocytes, it has been shown that Sgk1 or SGK1 is expressed in neurons in several diseases such as Parkinson's disease [65–67], Huntington's disease [68], amyotrophic lateral sclerosis (ALS) [67], and ischemia [69]. Moreover, in experimental animals, the administration of morphine [70], tumor necrosis factor- (TNF-) *α*, or transforming growth factor- (TGF-) *β* [71–74] has also been shown to upregulate Sgk1 expression in neurons. However, the molecular mechanism underlying the elevation of Sgk1 in neurons remains unknown [37].

**Figure 12:**
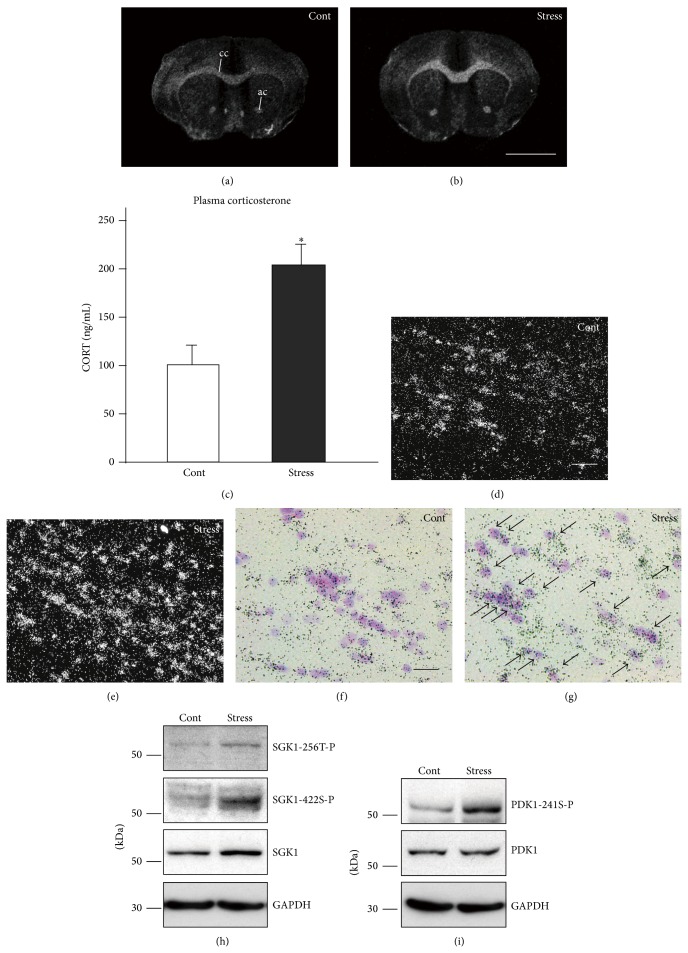
Repeated exposure to WIRS (chronic stress) upregulated SGK1 and SGK1 is activated by PDK1. (a, b)* In situ* hybridization images of* Sgk1* mRNA. Dark-field photomicrographs show the upregulation of* Sgk1* mRNA expression in the fiber tracts after repeated exposure to WIRS ((a) controls; (b) stress). cc, corpus callosum; ac, anterior commissure. Scale bar = 5 mm. (c) Alternation of plasma corticosterone 24 h after repeated WIRS. The results are expressed as the mean ± SEM of three independent experiments. ^*^ *P* < 0.05, *t*-test. (d, e) The CC region images of* Sgk1* mRNA* in situ* hybridization ((d) controls; (e) stress), respectively. Scale bar = 100 *μ*m. (f, g) Merged images of Nissl staining and* in situ* hybridization images of* Sgk1* mRNA. The distribution of cells expressing* Sgk1* mRNA in the CC of the control (d) and repeated WIRS-exposed mice (e) in bright-filed photomicrographs. Positive grains were concentrated in the oligodendrocytes. Scale bars = 50 *μ*m. (h, i) Western blot analysis shows SGK1 protein, its phosphorylation at positions T-256 (SGK1-256T-P) and S-422 (SGK1-422S-P), and the phosphorylation of PDK1 at position S-241 (PDK-241S-P) in the oligodendrocytes of the CC after repeated exposure to WIRS. (a)–(i) are adapted with permission from Miyata et al. [37].

Using the microarray technique, we have shown that* Sgk1* mRNA expression is consistently altered in the medial prefrontal cortex of chronically stressed mice. Furthermore, it has recently been shown that* Sgk1* mRNA expression is markedly increased in fiber tracts, such as the CC and anterior commissure, after repeated exposure to WIRS (Figures 12(a) and 12(b)) [37].

The increase in* Sgk1* occurs together with an increase of plasma corticosterone (Figure 12(c)), with* Sgk1* mRNA being almost exclusively localized to the oligodendrocytes (Figures 12(d)–12(g)). In addition, the stress-induced upregulation of* Sgk1* mRNA can be abolished by adrenalectomy [37]. Thus, these findings indicate that elevated levels of corticosterone after exposure to WIRS (activation of hypothalamic-pituitary-adrenal axis by stress) increases* Sgk1* expression in oligodendrocytes.* Sgk1* mRNA and SGK1 protein upregulation in fiber tracts has also been confirmed by qRT-PCR and western blotting analyses (Figure 12(h)) [37].

The next problem that should be addressed is whether upregulated SGK1 is in its active form or not. SGK activation is dependent on the phosphorylation of Thr(T)-256 in the activation loop and Ser(S)-422 in the hydrophobic motif near the C terminus [62–64]. As shown in Figure 12(h), the level of phosphorylated SGK1 is elevated in both sites in addition to the increased expression of SGK1. The Sgk1 mRNA expressions were concentrated in the oligodendrocytes and SGK1 protein expression was detected in the oligodendrocyte primary culture (unpublished data). Thus, SGK1 phosphorylation in the CC was thought to have occurred in the oligodendrocytes.

### 4.2. SGK1 Is Activated by PI3K-3-Phosphoinositide-Dependent Protein Kinase (PDK1), Which Is Phosphorylated by Activated PI3K

It has been demonstrated that in HEK293 cells (the glucocorticoid receptors, PDK1, SGK1, and NDGR1 are endogenously expressed in this cell line) phosphorylated phosphatidylinositol 3-kinase-3-phosphoinositide-dependent protein kinase (PDK1) is induced by phosphorylated phosphatidylinositol 3-kinase (PI3K), which in turn phosphorylates SGK1 [62–64]. Therefore, we examined whether PDK1 phosphorylation levels increase in the corpus callosum after repeated exposure to WIRS. Chronic stress elevates levels of phosphorylated PDK1 as well as that of phosphorylated SGK1 (Figures 12(h) and 12(i)). However, no notable alteration has been found with respect to PDK1 expression (Figure 12(i)).* In vitro* analysis using HEK293 cells has also demonstrated similar effects. Stimulating HEK293 cells with dexamethasone (DEX) has been shown to increase the level of PDK1 phosphorylation as well as that of SGK1 expression and phosphorylation; however, the same stimulation does not change the expression of PDK1 [37]. Interestingly, inhibition of PI3K signaling pathway by wortmannin (WORT; selective PI3K signal inhibitor) has been shown to abolish the dexamethasone-induced increase in phosphorylated PDK1, although expression levels of PDK1 are not affected [37]. Stimulating HEK293 cells with DEX can elevate both SGK1 expression and phosphorylated SGK1. Moreover, pretreatment with WORT has been shown to inhibit the DEX-induced increase in SGK1 phosphorylation but fails to inhibit the DEX-induced upregulation of SGK1 expression [37]. These findings indicate that repeated exposure to WIRS does not affect PDK1 production but elevates phosphorylation levels of PDK1.

These results show that in the oligodendrocytes, (1) repeated exposure to WIRS increases plasma corticosterone levels, (2) increased plasma corticosterone levels induce PDK1 phosphorylation, (3) upregulated phosphorylated PDK1 in turn phosphorylates SGK1, and (4) the increase in SGK1 expression is not induced by the activation of the PI3K pathway [37].

### 4.3. Upregulation of Phosphorylated SGK1 in Oligodendrocytes Increases N-myc Downstream-Regulated Gene 1 (NDRG1) Phosphorylation

**Figure 13:**
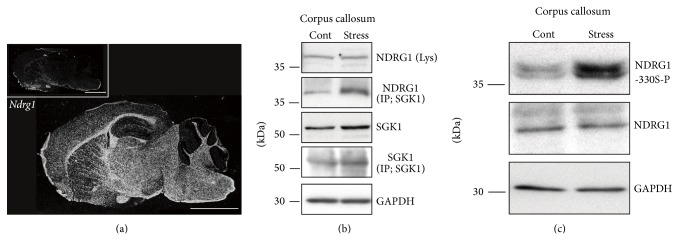
Repeated exposure to WIRS upregulates NDRG1 phosphorylation in the fiber tracts* via* SGK1 activation. (a)* In situ* hybridization images of* Ndrg1* mRNA. Dark-field photomicrographs show the distribution of* Ndrg1* mRNA-expressing cells in the mouse brain on the sagittal plane. Sections were hybridized with ^35^S-labeled antisense RNA probe for* Ndrg1* mRNA. As controls, adjacent sections were hybridized with ^35^S-labeled sense RNA probe (inset). Scale bar = 5 mm. (b) Immunoprecipitation and western blot analysis show that repeated exposure to WIRS elevated the interaction between SGK1 and NDRG1 (second column). However, NDRG1 expression did not increase in the CC (first column). (c) Western blot analysis shows that repeated exposure to WIRS elevated phosphorylated NDRG1 levels in the CC. (a)–(c) are adapted with permission from Miyata et al. [37].

It has been reported that several downstream targets of SGK1 in the brain are* N*-myc downstream-regulated gene 1 (NDRG1), NDRG2, Tau, Huntingtin, I*κ*B kinase *α* (IKK*α*), and p300 [68, 75–77]. Among them, NDRG1 has been shown to be localized in oligodendrocytes and is the substrate of SGK1 [75, 78]. Immunoprecipitation has revealed that repeated exposure to WIRS increases the interaction between SGK1 and NDRG1 in the CC (Figure 13(b)); however, this does not alter the expression level of either the protein or the mRNA (Figure 13(b)) [37]. In addition, it has been shown that NDRG1 is localized in oligodendrocytes (Figure 13(a)). These findings strongly suggest that an increase in the level of phosphorylated SGK1 in oligodendrocytes leads to stronger interactions between SGK1 and NDGR1, which, in turn, results in NDRG1 phosphorylation since NDRG1 is the substrate of SGK1 [37].

It has also been established that the phosphorylation of NDRG1 is achieved by active SGK1. S-330 in the NDRG1 protein is an important site that is phosphorylated by SGK1. The phosphorylation levels of NDRG1 in the CC have been demonstrated to be markedly elevated after repeated exposure to WIRS. Interestingly, however, protein expression levels in these experiments were not reported to be altered (Figure 13(c)). In the study using HEK293 cells* in vitro*, stimulating the cells with DEX was reported to increase the levels of phosphorylated NDRG1, total SGK1, and phosphorylated SGK1. Moreover, it has been shown that overexpression of the constitutively active form of SGK1 (SGK1-S422D; CA-SGK1) results in the phosphorylation of endogenous NDRG1 [37]. On the other hand, the overexpression of a constitutively kinase inactive form of SGK1 (SGK1-S422A; KI-SGK1) fails to phosphorylate NDGR1. Thus, the overexpression of CA-SGK1, but not KI-SGK1, can increase phosphorylation of overexpressed NDRG1 [37].

### 4.4. The Upregulation of SGK1 Phosphorylation and Subsequent Increase of NDRG1 Phosphorylation following Repeated Exposure to WIRS Increases the Expression of Adhesion Molecules in Oligodendrocytes

**Figure 14:**
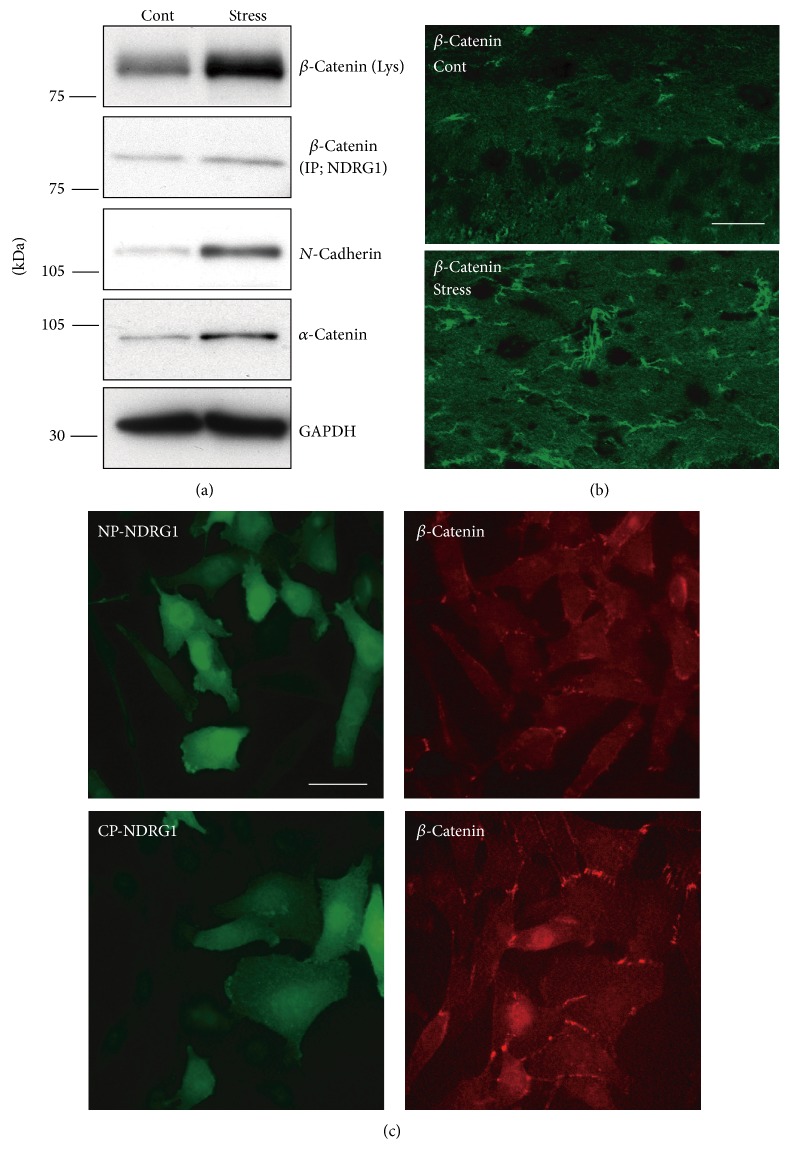
Repeated exposure to WIRS upregulates adhesion molecules expression levels in oligodendrocytes of the fiber tracts. (a) Immunoprecipitation and western blot analysis show that repeated exposure to WIRS elevated the interaction between NDRG1 and *β*-catenin (second panel) and that the expression levels of *β*-catenin,* N*-cadherin, and *α*-catenin were elevated in the corpus callosum. (b) Immunohistochemical analysis of *β*-catenin in the CC demonstrates increased labeling of the processes of the oligodendrocytes (i.e., greater number and intensity) in mice exposed to repeated WIRS. Scale bar = 50 *μ*m. (c) Immunocytochemical analysis of *β*-catenin in SK-N-SH cells overexpressing nonphosphorylated (NP-NDRG1) or constitutively phosphorylated NDRG1 (CP-NDRG1). Increased expression of *β*-catenin due to the overexpression of the CP-NDRG1 was mostly observed on the surfaces of the cell bodies and the processes of SK-N-SH cells. Scale bar = 20 *μ*m. (a)–(c) are adapted with permission from Miyata et al. [37].

Expression levels of the main adhesion molecules, such as* N*-cadherin, *α*-catenin, and *β*-catenin, which comprise adherens junctions, have been found to increase in the CC after repeated exposure to WIRS (Figure 14(a)). Moreover, immunohistochemical analysis has exhibited that repeated exposure to WIRS results in an increase in the number of oligodendrocyte processes labeled by adhesion molecules in the CC, as well as thicker processes than those of controls (Figure 14(b)). Furthermore, these processes could be traced for longer distances than those of controls.

An immunoprecipitation study has shown that NDRG1 coimmunoprecipitates with *β*-catenin but not with* N*-cadherin or *α*-catenin (Figure 14(a)). In addition, the coimmunoprecipitation of NDRG1 with *β*-catenin has been shown to increase in the lysates of the CC after repeated exposure to WIRS (Figure 14(a)).

In SK-N-SH cells (one of the human neuroblastoma cell lines) expressing NDRG1-S330A (constitutively nonphosphorylated NDRG1; NP-NDRG1), weak and diffusely distributed staining can be observed for each of the adhesion molecules throughout the cytoplasm, including the cell membrane (Figure 14(c), upper panels). In contrast, SK-N-SH cells that express constitutively phosphorylated NDRG1 (NDRG1-S330D; CP-NDRG1) exhibit more pronounced immunoreactivity for each of the three adhesion molecules, particularly at the membrane surface (Figure 14(c), lower panels).

Overexpression of CP-NDRG1 has been shown to increase the expression of* N*-cadherin, *α*-catenin, and *β*-catenin, while overexpression of NP-NDRG1 has been shown to have no noticeable effect on the expression levels of these molecules [37]. In addition, the coimmunoprecipitation of NDRG1 with *β*-catenin has been revealed to increase after CP-NDRG1 overexpression [37].

Thus, the findings above reveal that (1) repeated exposure to WIRS increases the expression of phosphorylated NDRG1 and the interaction between phosphorylated NDRG 1 and *β*-catenin; (2) increased phosphorylation of NDRG1 expression upregulates the expression of * N*-cadherin, *α*-catenin, and *β*-catenin; and (3) increased amounts of these adhesion molecules localize to the cell membrane.

### 4.5. Repeated Exposure to WIRS Causes Morphological Alterations to Oligodendrocytes in the CC

**Figure 15:**
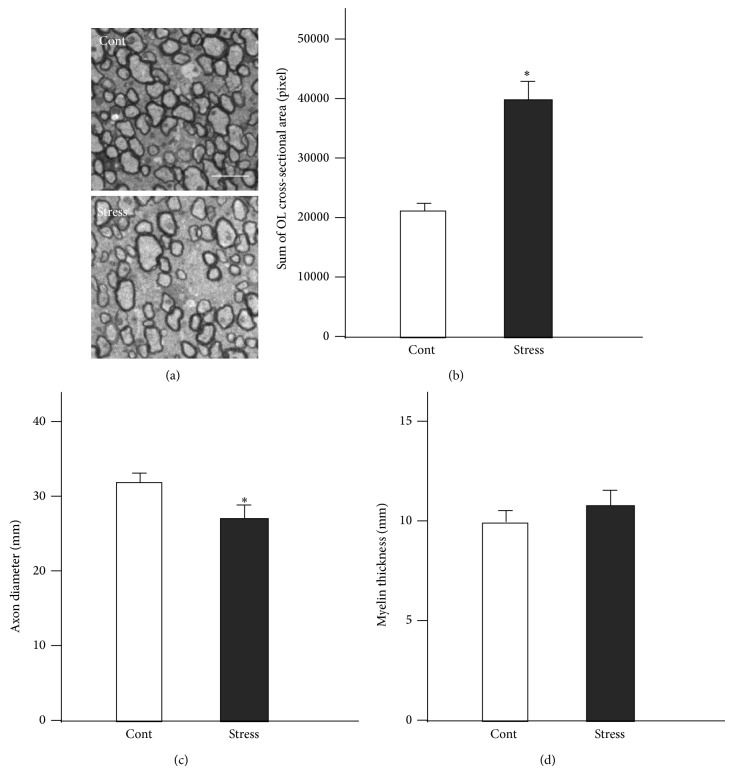
Repeated exposure to WIRS causes morphological alterations in oligodendrocytes in the CC. (a) Representative transverse electron micrographs of the CC from control (upper panel of (a)) and repeated WIRS-exposed mice (lower panels of (a)). Scale bar = 2 *μ*m. (b) Results of the quantification of the sum of oligodendrocytes in the cross-sectional area. The results are expressed as the mean ± SEM of 3 independent experiments. ^*^ *P* < 0.05, *t*-test. (c, d) The distributions of the axon diameters (c) and myelin thicknesses (d) of the CC in the control and repeated WIRS-exposed mice were assessed. The results are expressed as the mean ± SEM of 3 independent experiments. ^*^ *P* < 0.05, *t*-test. (a)–(d) are adapted with permission from Miyata et al. [37].

Although no clear morphological alteration in the CC has been reported after repeated exposure to WIRS at the light microscopic level [37], electron microscopic analyses clearly show morphological changes in oligodendrocytes in the CC after the same stimulation (Figures 15(a) and 15(b)) [37]. In control mice, a number of myelinated fibers compactly gather in the CC (Figure 15(a), cont). However, following repeated exposure to WIRS, the interfibrial space, which is occupied almost entirely by the oligodendrocytes, markedly increases (Figure 15(a), stress). Statistical analyses revealed the interfibrial space in WIRS-exposed mice to be twice as large as that in control mice (Figure 15(b)). In addition, the average diameter of the nerve fibers was reported to be smaller in the CC of WIRS-exposed mice than those of controls (Figure 15(c)). However, in this study, there was no significant change in the thickness of myelin in the CC of WIRS-exposed mice compared to controls (Figure 15(d)). Thus, these observations demonstrate that repeated exposure to WIRS causes excess arborization of oligodendrocyte processes.

### 4.6. Activation of the PDK1-SGK1-NDRG1 Pathway by Repeated Exposure to WIRS Increases Oligodendrocyte Volume in the CC

**Figure 16:**
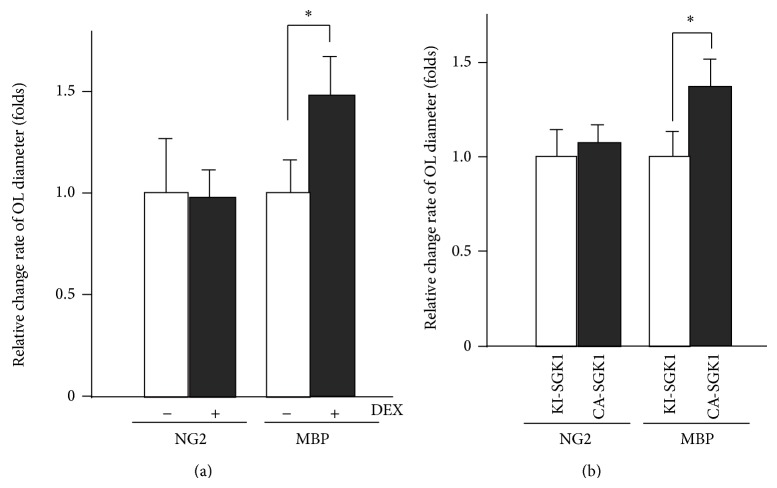
Activation of the SGK1-NDRG1 pathway also causes morphological changes in primary cultured oligodendrocytes. (a) Morphology of primary cultured oligodendrocytes treated with (+DEX) ((a) right column) or without DEX (−DEX) for 2 days. (b) Overexpression of the constitutively active form of SGK1 (CA-SGK1) or kinase inactive form of SGK1 (KI-SGK1). (a, b) Morphometric measurements of oligodendrocyte diameters were performed using ImageJ software. The results are expressed as the mean ± SEM of 3 independent experiments. ^*^ *P* < 0.05, *t*-test. (a) and (b) are adapted with permission from Miyata et al. [37].

To determine whether SGK1-NDRG1 cascade activation affects the morphology of OPCs or mature oligodendrocytes, DEX can be added to culture medium of both mature and immature oligodendrocytes. Mature and immature oligodendrocytes can then be identified by using the specific markers, MBP and NG2, respectively. The addition of DEX has been found to result in an approximately 1.5-fold increase in the diameter of the oligodendrocytes labeled by MBP compared to untreated oligodendrocytes (Figure 16(a)) [37]. Furthermore, CA-, KI-SGK1 and CP-, NP-NDRG1 were overexpressed in primary OPCs and mature oligodendrocytes by adenovirus infection. Overexpression of CA-SGK1 and CP-NDRG1 in oligodendrocytes has the same effect as repeated exposure to WIRS. In contrast, overexpression of CN-SGK1 and NP-NDRG1 does not induce morphological changes in oligodendrocytes; however, these inactive forms do increase oligodendrocyte diameter (about 1.4-fold increase for SGK1 and NDRG1) (Figure 16(b)) [37].

Furthermore, it should be stressed that the effects of DEX, as well as the active forms of SGK1 and NDRG1, are not found in OPCs labeled with NG2. These results indicate that the HPA axis-SGK-NDRG1 signaling pathway strongly impacts mature oligodendrocytes causing morphological alterations, whereas this is not the case in OPCs (Figure 16) [37].

**Figure 17:**
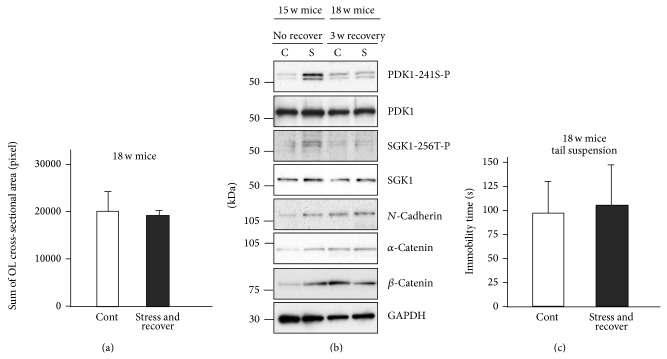
Activation of the PDKI-SGKI-NDRG1-adhesion molecule pathway returns to the control level after 3 weeks of recovery. (a) Quantification of the sum of the cross-sectional areas of the oligodendrocytes is measured from transverse electron micrographs of the CC of no-stress controls (18-week-old mice) (Cont) and after 3 weeks of recovery after exposure to WIRS (18-week-old mice) (stress and recover). Morphometric measurements were made using ImageJ software. The results are expressed as the mean ± SEM of 3 independent experiments. ^*^ *P* < 0.05, *t*-test. (b) Western blot analysis of the CC of mice exposed to repeated WIRS (15-week-old mice) and after 3 weeks of recovery (18-week-old mice). (c) Effects of a chronic stress, that is, 3 weeks of recovery, on mouse behavior. After 3 weeks of recovery mice (18-week-old mice) were discontinued showing no significant difference in the tail-suspension test compared to the control mice (18-week-old mice). The results are expressed as the mean ± SEM of 3 independent experiments. ^*^ *P* < 0.05, *t*-test. (a)–(c) are adapted with permission from Miyata et al. [37].

In addition, it should be noted that the increased immobility time and interfibrial space in the CC following repeated exposure to WIRS return to control levels after 3 weeks of recovery (Figures 17(a) and 17(c)). Furthermore, activation of the PDK1-SGK1-NDRG1-adhesion molecule pathway following repeated exposure to WIRS can also return to the basal levels after 3 weeks of recovery (Figure 17(b), 18 w mice).

### 4.7. Functional Significance of the Activation of PDK1-SGK1-NDRG1-Adhesion Molecules in Oligodendrocytes following Stress

**Figure 18:**
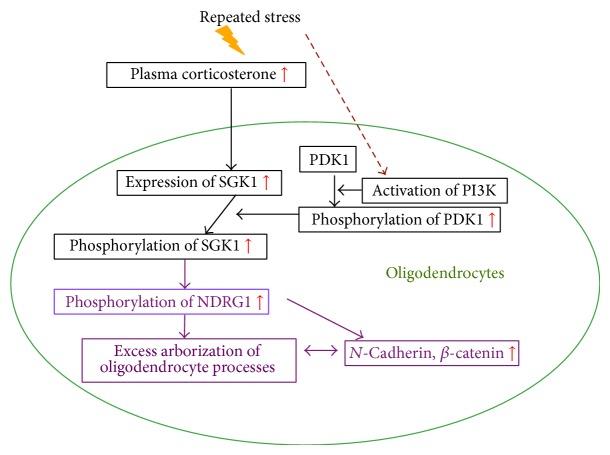
Activation of HPA axis-PDK1-SGK1-NDRG1-adhesion molecules by repeated stress induces excess arborization of the oligodendrocyte processes.

It can be concluded that repeated stress activates the PDK1-SGK1-NDRG1-adhesion molecule pathway via an increase in plasma corticosterone levels, and that the activation of this signaling pathway causes excess arborization of oligodendrocyte processes. Furthermore, the abnormality in oligodendrocytes is related to depressive-like symptoms (Figure 18) [37].

Overexpression of CA-SGK1 and CP-NDRG1 increases the size of oligodendrocytes. Thus, the activation of the PDK1-SGK1-NDRG1 pathway by repeated stress directly induces the increase in the cellular volume of oligodendrocytes.

The upregulation of Sgk1 in oligodendrocytes after cortical injury was first reported by Imaizumi et al. [79] and was confirmed by Hollister et al. [80] following hippocampal injury. Since brain injury does not induce elevations of plasma corticosterone, a mechanism other than activation of the HPA pathway is involved in the upregulation of SGK. Furthermore, in these cases, no increase in oligodendrocyte volume was detected; thus, the downstream effects of increased SGK1 level following cortical injury may be different from those caused by repeated stress.

Since NDRG1 is not thought to present in neurons [78], it is likely that other molecules may be involved downstream of SGK1. In any case, the molecular mechanisms and functional significance underlying the elevation of Sgk1 in neurons remains unknown.

### 4.8. Conclusion about SGK1 Functions in Oligodendrocytes in the Pathogenesis of MDD

Taken together, these findings suggest that repeated stress induces a disturbance of the CC* via* activation of the PDK1-SGK1-NDRG1-adhesion molecule pathway. The functional significance of the enlargement or excess arborization of oligodendrocytes occupying the interfibrial space in the CC after stress exposure is not clear at present. However, repeated stress remarkably reduces MBP expression. Accordingly, it is probable that abnormal swelling of the cytoplasm in oligodendrocytes impairs neurotransmission or interactions among nerve fibers in the CC [81, 82].

## 5. Conclusion

Compromised white matter/myelin integrity has been reported not only in neuropsychiatric diseases including SZ and MDD [28, 39–41, 50–52] but also in animal models of MDD [37]. We previously found that DISC1 dysfunction can impair differentiation of oligodendrocytes [19], and an increase of rare nonsynonymous mutations in patients with SZ has been recently reported [54]. Furthermore, using brain imaging and postmortem evaluations of the human brain, patients with MDD have been found to exhibit abnormalities in white matter and/or oligodendrocytes [83]. Interestingly, stress exposure in animal models can decrease the number of oligodendrocytes in the cortex and amygdala [84], suggesting potential links between disturbed myelination and MDD. A primary goal of future research is to understand how excessive arborization of oligodendrocyte processes contributes to the molecular pathogenesis of SZ and MDD. Therefore, it will be intriguing to examine whether DISC1, DBZ, and SGK1 play roles in the compromised white matter/myelin integrity that have been reported in patients with SZ and MDD.
